# dynGENIE3: dynamical GENIE3 for the inference of gene networks from time series expression data

**DOI:** 10.1038/s41598-018-21715-0

**Published:** 2018-02-21

**Authors:** Vân Anh Huynh-Thu, Pierre Geurts

**Affiliations:** Department of Electrical Engineering and Computer Science, University of Liège, 4000 Liège, Belgium

## Abstract

The elucidation of gene regulatory networks is one of the major challenges of systems biology. Measurements about genes that are exploited by network inference methods are typically available either in the form of steady-state expression vectors or time series expression data. In our previous work, we proposed the GENIE3 method that exploits variable importance scores derived from Random forests to identify the regulators of each target gene. This method provided state-of-the-art performance on several benchmark datasets, but it could however not specifically be applied to time series expression data. We propose here an adaptation of the GENIE3 method, called dynamical GENIE3 (dynGENIE3), for handling both time series and steady-state expression data. The proposed method is evaluated extensively on the artificial DREAM4 benchmarks and on three real time series expression datasets. Although dynGENIE3 does not systematically yield the best performance on each and every network, it is competitive with diverse methods from the literature, while preserving the main advantages of GENIE3 in terms of scalability.

## Introduction

Gene regulatory networks (GRNs) define the ensemble of interactions among genes that govern their expression. The elucidation of GRNs is crucial to understand the functioning and pathology of organisms, and remains one of the major challenges of systems biology. Since the advent of high-throughput technologies, computational approaches have been proposed to infer GRNs from the measurement of gene expressions in various conditions using statistical inference or machine learning techniques. While network inference methods have reached some maturity over the years, their performance on real datasets remains far from optimal and calls for the permanent improvement of existing methods.

Measurements about genes that are exploited by these methods are typically available in two forms: static steady-state expression vectors, obtained by applying a perturbation to the system under study and measuring gene expressions once the system has reached some equilibrium point, or time series expression data, measuring the temporal evolution of gene expressions over several time points following the perturbation. Steady-state expression data are plethoric for many organisms. They however offer limited information regarding the dynamics of gene regulation, which limits the performance of network inference methods when they only exploit such data. Time series data on the other hand are intrinsically much more informative about the dynamics and should in principle make the inference more effective than steady-state data. In particular, time series data allow to infer causal relationships among genes, by analysing the cascade of expression changes across time. Unfortunately, collecting time series data poses several important technical and design issues that make such data very scarce^1^. One issue comes from the fact that expression data are currently mainly obtained using microarray or RNA-seq technologies, which both measure the gene expressions in populations of cells. Inaccurate measurements can thus occur if the cells are not synchronised at the different sampling time points. A second important issue is the choice of the sampling time points. The high cost of genomic experiments usually prevents a dense sampling over a long time period, and it may be difficult to choose the correct time points that will allow to capture all the expression changes. Dealing with the scarcity in time series expression data is an important challenge for network inference methods and this calls also for methods that can exploit jointly both steady-state and time series data.

Mostly two families of methods have been explored in the literature to solve the GRN inference problem: model-free and model-based methods. Model-free methods infer the network by directly estimating the gene dependencies from the data through more or less sophisticated statistical or machine learning-based analyses^2–6^. These methods typically have good scalability, enabling reconstructions of networks of thousands of genes, and have consistently achieved state-of-the-art reconstruction performance in comparative evaluations^7^. On the other hand, model-based methods first define a quantitative dynamical model of the system, for example using differential equations^8^ or auto-regressive models^9^, and then infer the network by learning the parameters of this model from observed time series data. Model-based methods are rather computationally intensive and their parametric nature usually implies very stringent assumptions about the dynamics (e.g. linearity). These methods have nevertheless some appealing properties that model-free methods do not have: they have clearly defined semantics in terms of the underlying dynamical system properties, which makes them more interpretable than model-free methods. Most importantly, model-based methods can be used for simulating and predicting the dynamical system behaviour under perturbations.

In our previous work, we proposed GENIE3, a model-free method that infers networks from steady-state expression data^6^. This method exploits variable importance scores derived from Random forests^10^ to identify the regulators of each target gene. The main properties of this method are its fully non-parametric nature, its good scalability, and its ease of use. GENIE3 was the best performer of the DREAM4 *Multifactorial Network* challenge and the DREAM5 *Network Inference* challenge^7^, and has since been shown to be competitive with several other methods in several independent studies (e.g.^11,12^). Motivated by the good performance of GENIE3 on steady-state data, the aim of this paper is to evaluate GENIE3 and a new variant of GENIE3, when they are applied for the analysis of time series data and for the joint analysis of steady-state and time series data. The proposed variant for time series data, called dynGENIE3 (for dynamical GENIE3), is based on a semi-parametric model, in which the temporal evolution of each gene expression is described by an ordinary differential equation (ODE) and the transcription function in each ODE is learned in the form of a non-parametric Random forest model. The regulators of each target gene are then identified from the variable importance scores derived from the corresponding Random forest model.

Several experiments are carried out on artificial and real datasets to assess the performance of GENIE3 and dynGENIE3. While dynGENIE3 consistently outperforms GENIE3 on the artificial data, the relative performances of the two methods become very data-dependent when they are applied to real data. In addition, our experiments show that, even though dynGENIE3 does not systematically reach the best performance in every setting, it is nevertheless very competitive with existing methods from the literature.

To summarise, dynGENIE3 is a highly scalable network inference method able to exploit time series and steady-state data jointly. It consistently yields good performance on diverse artificial and real networks. On the DREAM4 networks, it is only outperformed by CSI^13^, a Bayesian inference method based on Gaussian processes. CSI has however the major drawback of being very computationally intensive, with respect to the number of observations and the number of candidate regulators (more details can be found in the “Related works” section).

The present work supersedes the time series extension of GENIE3 that we proposed previously^14^ and that was applied for the inference of the GRN underlying the drought response of common sunflower^15^.

## Methods

### Problem definition

Let *D*_*TS*_ and *D*_*SS*_ be two expression datasets. The first dataset *D*_*TS*_, called the *time series dataset*, contains the expression levels of *p* genes, measured at *N* time points following a perturbation of the network:

1 $${D}_{TS}=\{{\bf{x}}({t}_{1}),{\bf{x}}({t}_{2}),\ldots ,{\bf{x}}({t}_{N})\},$$

where ${\bf{x}}({t}_{k})\in {{\mathbb{R}}}^{p}$, *k* = 1, …, *N* is a vector containing the gene expression values at the *k*-th time point:

2 $${\bf{x}}({t}_{k})={({x}_{1}({t}_{k}),{x}_{2}({t}_{k}),\ldots ,{x}_{p}({t}_{k}))}^{{\rm{{\rm T}}}}.$$

The other dataset *D*_*SS*_, called the *steady*-*state dataset*, contains the expression levels of the same *p* genes, measured in *M* experimental conditions once the system has reached some equilibrium point:

3 $${D}_{SS}=\{{\bf{x}}({e}_{1}),{\bf{x}}({e}_{2}),\ldots ,{\bf{x}}({e}_{M})\},$$

where ${\bf{x}}({e}_{k})\in {{\mathbb{R}}}^{p}$, *k* = 1, …, *M* is a vector containing the expression values at steady-state of the *p* genes in the *k*-th condition:

4 $${\bf{x}}({e}_{k})={({x}_{1}({e}_{k}),{x}_{2}({e}_{k}),\ldots ,{x}_{p}({e}_{k}))}^{{\rm{{\rm T}}}}.$$

The goal is to exploit *D*_*TS*_, possibly together with *D*_*SS*_, in order to assign weights *w*_*i*,*j*_ ≥ 0, (*i*, *j* = 1, …, *p*) to putative regulatory links from any gene *i* to any gene *j*, with the aim of assigning the largest weights to links that correspond to actual regulatory interactions. Note that in this article, we leave open the problem of automatically choosing a threshold on the weights to obtain a practical network and focus on providing a ranking of the regulatory links.

### The GENIE3 framework

#### The original GENIE3 method for steady-state data

The GENIE3 method^6^ treats the network inference problem as *p* feature selection problems, each feature selection problem consisting in recovering the regulators of a given gene. The method was originally designed to exploit steady-state data and makes the assumption that the expression of each gene *j* in a given condition is a function of the expression levels of the other genes in the same condition:

5 $${x}_{j}({e}_{k})={f}_{j}({{\bf{x}}}_{-j}({e}_{k}))+{\varepsilon }_{k},\forall j,k,$$

where **x**_−*j*_ denotes the vector containing the expression levels of all the genes except gene *j* and *ε*_*k*_ is a random noise. GENIE3 further makes the assumption that the function *f*_*j*_ only exploits the expression in **x**_−*j*_ of the genes that are direct regulators of gene *j*, i.e. genes that are directly connected to gene *j* in the targeted network. Recovering the regulatory links pointing to gene *j* thus amounts to finding those genes whose expression is predictive of the expression of gene *j*.

The GENIE3 procedure works as follows:

- For *j* = 1 to *p*:
  1. Generate the learning sample of input-output pairs for gene *j*:
    6 $$L{S}_{SS}^{j}=\{({{\bf{x}}}_{-j}({e}_{k}),{x}_{j}({e}_{k})),k=1,\ldots ,M\mathrm{\}}.$$
  2. Learn *f*_*j*_ from $L{S}_{SS}^{j}$ and use a feature ranking technique to compute confidence levels *w*_*i*,*j*_(*i* ≠ *j*), *i* = 1, …, *p*, for all the genes except gene *j*.
- Use *w*_*i*,*j*_ as weight for the regulatory link *i* → *j*.

Note that when a set of candidate regulators (e.g. known transcription factors) is given, the input genes in $L{S}_{SS}^{j}$ can be restricted to these candidate regulators only. In that case, the weights *w*_*i*,*j*_ such that gene *i* is not a candidate regulator are set to zero.

#### dynGENIE3 for time series data

GENIE3 can be applied to time series data in a naive way, by regarding the different time points as independent steady-state conditions. An alternative solution is to modify the procedure in order to take into account the dependence between the time points. The dynamical variant of GENIE3 (dynGENIE3) assumes that the expression level of gene *j* is modelled through the following ordinary differential equation (ODE):

7 $$\frac{{\rm{d}}\,{x}_{j}(t)}{{\rm{d}}t}=-{\alpha }_{j}{x}_{j}(t)+{f}_{j}({\bf{x}}(t)),\forall j,$$

where we assume that the transcription rate of *x*_*j*_ is a (potentially non-linear) function *f*_*j*_ of the expression levels of the *p* genes (possibly including the gene *j* itself) and *α*_*j*_ is a parameter specifying the decay rate of *x*_*j*_.

The ODE (7) has the following finite approximation:

8 $$\begin{array}{rcl}\frac{{x}_{j}({t}_{k+1})-{x}_{j}({t}_{k})}{{t}_{k+1}-{t}_{k}}+{\alpha }_{j}{x}_{j}({t}_{k}) & = & {f}_{j}({\bf{x}}({t}_{k})),\\ k & = & 1,\ldots ,N-1,\end{array}$$

and the function *f*_*j*_ can thus be learned using the following learning sample:

9 $$L{S}_{TS}^{j}=\{({\bf{x}}({t}_{k}),\frac{{x}_{j}({t}_{k+1})-{x}_{j}({t}_{k})}{{t}_{k+1}-{t}_{k}}+{\alpha }_{j}{x}_{j}({t}_{k})),k=1,\ldots ,N-1\}.$$

Note that this procedure allows the incorporation of multiple time series experiments by learning the transcription function *f*_*j*_ from the concatenation of the learning samples $L{S}_{TS}^{j}$ respectively generated from the different experiments.

The ODE model (7) and its finite approximation (8) have been used successfully by the Inferelator method for modelling and inferring gene regulatory interactions^16–18^. A more detailed comparison with this method is provided in the “Related works” section.

It is interesting to note that when the time interval *t*_*k*+1_ − *t*_*k*_ is constant $\forall k$ and ${\alpha }_{j}=\frac{1}{{t}_{k+1}-{t}_{k}}$, the equation (8) simplifies to:

10 $${x}_{j}({t}_{k+1})=({t}_{k+1}-{t}_{k}){f}_{j}({\bf{x}}({t}_{k}))={f^{\prime} }_{j}({\bf{x}}({t}_{k})),$$

which is equivalent to a time-lagged version of the original GENIE3 method^14^.

#### dynGENIE3 for both time series and steady-state data

At steady-state, $\frac{{\rm{d}}{x}_{j}(t)}{{\rm{d}}t}=0$ and the equation (7) becomes:

11 $${\alpha }_{j}{x}_{j}(t)={f}_{j}({\bf{x}}(t)),\forall j.$$

The learning sample *LS *^*j*^ used to learn the function *f*_*j*_ can thus be obtained by concatenating the two types of data:

12 $$L{S}^{j}=L{S}_{TS}^{j}\cup L{S}_{SS}^{j},$$

where $L{S}_{TS}^{j}$ (resp. $L{S}_{SS}^{j}$) is the learning sample generated from the time series (resp. steady-state) data:

13 $$\begin{array}{rcl}L{S}_{TS}^{j} & = & \{({\bf{x}}({t}_{k}),\frac{{x}_{j}({t}_{k+1})-{x}_{j}({t}_{k})}{{t}_{k+1}-{t}_{k}}+{\alpha }_{j}{x}_{j}({t}_{k})),k=1,\ldots ,N-1\}.\\ L{S}_{SS}^{j} & = & \{({\bf{x}}({e}_{k^{\prime} }),{\alpha }_{j}{x}_{j}({e}_{k^{\prime} })),k^{\prime} =1,\ldots ,M\}.\end{array}$$

#### Tree-based methods

In GENIE3 and dynGENIE3, the function *f*_*j*_ is learned in the form of an ensemble of regression trees. Regression trees split the data samples with binary tests based each on one input variable, trying to reduce as much as possible the variance of the output variable in the resulting subsets of samples. Candidate splits for numerical variables compare the input variable values with a threshold that is determined during the tree growing. Single trees are usually very much improved by ensemble methods that average the predictions of several trees. For example, in a Random forest ensemble each tree is built from a bootstrap sample of the original learning sample and at each test node *K* variables are selected at random among all the input variables before determining the best split^10^.

#### Variable importance measure

It is possible to compute, from a tree model, variable importance scores assessing the relevance of the different input features for predicting the output. In our experiments, we consider the Mean Decrease Impurity measure^19^ that computes, at each test node ${\mathscr{N}}$, the total reduction of the variance of the output variable due to the split:

14 $$I({\mathscr{N}})=\#S.{\rm{Var}}(S)-\#{S}_{t}.{\rm{Var}}({S}_{t})-\#{S}_{f}.{\rm{Var}}({S}_{f}),$$

where *S* denotes the set of samples that reach node ${\mathscr{N}}$, *S*_*t*_ (resp. *S*_*f*_) denotes its subset for which the test is true (resp. false), Var(.) is the variance of the output variable in a subset, and # denotes the cardinality of a set of samples. Given one regression tree, the overall importance *w* of one variable is computed by summing the *I* values (14) of all the tree nodes where this variable is used to split. Those variables that are not selected at all obtain a zero value of their importance, and those that are selected close to the root node typically obtain high scores. For an ensemble of trees, the importance *w* is averaged over the different trees.

#### Regulatory link ranking

The sum of the importance scores *w*_*i*,*j*_ of all the input features for one tree is usually very close to the initial total variance of the output. We thus have:

15 $$\sum _{i=1}^{p}\,{w}_{i,j}\approx {N}_{S}.{{\rm{Var}}}_{j}(S),\forall j$$

where *S* is the learning sample from which the tree was built (i.e. a bootstrap sample of *LS *^*j*^ for the Random forest method), *N*_*S*_ is the size of *S*, and Var_*j*_(*S*) is the variance of the target gene *j* estimated in *S*. As a consequence, if we trivially use the scores *w*_*i*,*j*_ to order the regulatory links, this is likely to introduce a positive bias for the regulatory links directed towards the genes whose expression levels vary the most. To avoid this bias, we normalize each importance score *w*_*i*,*j*_ by the total variance that is explained by the putative regulators (excluding self-interactions):

16 $$\begin{array}{l}{w}_{i,j}\leftarrow \frac{{w}_{i,j}}{{\sum }_{i\ne j}^{p}{w}_{i,j}},\forall i\ne j,\\ {w}_{j,j}=0.\end{array}$$

This normalization implies that the importance scores inferred from different models predicting different gene expressions are comparable.

#### Decay rate values

In the ODE model (7), the kinetic parameter *α*_*j*_, *j* = 1, …, *p* represents the decay rate of the expression of gene *j*. Its value may be retrieved from the literature, since there exist many studies that experimentally measure the mRNA decay rates in different organisms. However, when such information is not available or when dealing with simulated data, we use the same approach as in the Jump3 method^20^. In this method, the value of the decay rate *α*_*j*_ is estimated directly from the observed expression **x**_*j*_, by assuming an exponential decay ${e}^{-{\alpha }_{j}t}$ between the highest and lowest values of **x**_*j*_. In the remaining of this paper, the *α*_*j*_ values estimated using this method will be called the “data-derived” values.

#### Availability

Python, MATLAB and R implementations of dynGENIE3 are available at http://www.montefiore.ulg.ac.be/˜huynh-thu/dynGENIE3.html.

### Related works

Like dynGENIE3, many network inference approaches for time series data are based on an ODE model of the type (7) ^8,21^. These methods mainly differ in the terms present in the right-hand side of the ODE (such as decay rates or the influence of external perturbations), the mathematical form of the models *f*_*j*_, the algorithm used to train these models, and the way a network is inferred from the resulting models. dynGENIE3 adopts the same ODE formulation as in the Inferelator approach^16^: each ODE includes a term representing the decay of the target gene and the functions *f*_*j*_ take as input the expression of all the genes at some time point *t*. In the specific case of dynGENIE3, the functions *f*_*j*_ are represented by ensembles of regression trees, which are trained to minimize the least-square error using the Random forest algorithm, and a network is inferred by thresholding variable importance scores derived from the Random forest models. Like for the standard GENIE3, dynGENIE3 has a reasonable computational complexity, which is at worst *O*(*prN* log *N*), where *p* is the total number of genes, *r* is the number of candidate regulators and *N* is the number of observations.

In comparison, most methods in the literature (including Inferelator) assume that the models *f*_*j*_ are linear and train these models by jointly maximizing the quality of the fit and minimizing some sparsity-inducing penalty (e.g. using a L1 penalty term or some appropriate Bayesian priors). After training the linear models, a network can be obtained by analysing the weights within the models, several of which having been enforced to zero during training. In contrast to these methods, dynGENIE3 does not make any prior hypothesis about the form of the *f*_*j*_ models. This is an advantage in terms of representational power but this could also result in a higher variance, and therefore worse performance because of overfitting, especially when the data is scarce. A few methods also exploit non-linear/non-parametric models within a similar framework, among which Jump3^20^, OKVAR-Boost^22^ and CSI^13^. Like dynGENIE3, Jump3 incorporates a (different) dynamical model within a non-parametric, tree-based approach. In the model used by Jump3, the functions *f*_*j*_ represent latent variables, which necessitated the development of a new type of decision tree, while Random forests can be applied as such in dynGENIE3. One drawback of Jump3 is its high computational complexity with respect to the number *N* of observations, being *O*(*N*^4^) in the worst-case scenario. Moreover, Jump3 can not be used for the joint analysis of time series and steady-state data. OKVAR-Boost jointly represents the models *f*_*j*_ for all genes using an ensemble of operator-valued kernel regression models trained using a randomized boosting algorithm. The network structure is then estimated from the resulting model by computing its Jacobian matrix. One of the drawbacks of this method with respect to dynGENIE3 is that it requires to tune several meta-parameters. The authors have nevertheless proposed an original approach to tune them based on a stability criterion. Finally, CSI is a Bayesian inference method that learns the *f*_*j*_ models in the form of Gaussian processes. Since learning Gaussian processes does not embed any feature selection mechanism, network inference is performed in CSI by a combinatorial search through all the potential sets of regulators for each gene in turn, and constructing a posterior probability distribution over these potential sets of regulators. As a consequence, the complexity of the method is *O*(*pN*^3^*r*^* d*^/(*d* − 1)!), where *d* is a parameter defining the maximum number of regulators per gene^8^. Its high complexity makes CSI unsuitable when the number of candidate regulators (*r*) or the number of observations (*N*) is too high. Supplementary Table S1 compares the running times of dynGENIE3 and CSI for different datasets. The most striking difference is observed when inferring the DREAM4 100-gene networks. While dynGENIE3 takes only several minutes to infer one network, CSI can take more than 48 hours *per* target gene. The CSI algorithm can be parallelised over the different target genes (like dynGENIE3), but even in that case the computational burden remains an issue when inferring large networks containing thousands of genes and hundreds of transcription factors (such as the *E*. *coli* network).

### Performance metrics

GENIE3 and dynGENIE3 both provide a ranking of the regulatory links from the most confident to the least confident. To evaluate such a ranking independently of the choice of a specific threshold, we use the precision-recall (PR) curve and the area under this curve (AUPR), as suggested by the DREAM consortium^7,23–25^. The PR curve plots, for different thresholds on the weights of the links, the proportion of true positives among all the predictions (precision) versus the percentage of true positives that are retrieved (recall). A perfect ranking, i.e. a ranking where all the positives are located at the top of the list, yields an AUPR equal to one, while a random ranking results in an AUPR close to the proportion of positives in the true network.

For the DREAM4 networks (see below for the data description), we used the “AUPR score”, as proposed by the challenge organizers, to aggregate the AUPRs obtained for *n* different networks:

17 $${\rm{AUPR}}\,{\rm{score}}=-\frac{1}{n}\,\sum _{i=1}^{n}\,{\mathrm{log}}_{10}\,{p}_{AUPR}^{(i)},$$

where ${p}_{AUPR}^{(i)}$ is the probability for the *i*-th network that a given or larger AUPR is obtained by a random ranking of the putative edges. This probability is estimated from 100,000 random edge rankings. A higher AUPR score thus indicates a better overall performance over the *n* networks.

## Results

We first evaluated the performances of GENIE3 and dynGENIE3 on the simulated data of the DREAM4 *In Silico Network* challenge (note that this is a different challenge than the DREAM4 *Multifactorial Network* challenge where GENIE3 was deemed the best performer). We then applied the methods to three real expression datasets related to different organisms (*Saccharomyces cerevisiae*, *Drosophila melanogaster* and *Escherichia coli*). Supplementary Table S1 summarizes the total numbers of samples, genes and transcription factors in each dataset. Unless otherwise stated, in all our experiments ensembles of *T* = 1000 trees were grown and the main parameter *K* of the Random forest algorithm was set to the number of input candidate regulators.

### DREAM4 *in silico* networks

The goal of the DREAM4 *In Silico Network* challenge was to recover 5 networks of 10 genes and 5 networks of 100 genes, from both time series and steady-state data. Each time series experiment consisted in a perturbation that is applied to the network at time *t* = 0 and is removed after 10 time points, making the system return to its original state. Each time series contains noisy gene expressions levels that were sampled at 21 time points, with equal time intervals of 50 time units. The steady-state data contain the gene expression levels in various experimental conditions (wild-type, single gene knockouts, single gene knockdowns and multifactorial perturbations).

#### Network inference from time series data

We first compared GENIE3 and dynGENIE3 to various network inference algorithms, using only the time series data. Among the competitors are algorithms based on decision trees (Jump3^20^), pairwise mutual information (CLR^4^ and its time-lagged variant tlCLR^26^), dynamic Bayesian networks (G1DBN^27^ and VBSSM^28^), ordinary differential equations (tlCLR/Inferelator pipeline^17^ and TSNI^29^), non-linear dynamical systems (GP4GRN^30^, CSI^13^ and OKVAR-Boost^22^) and Granger causality (GCCA^31^). Since the expression data are here simulated, we can not use known biology in order to set the values of the degradation rates *α*_*j*_ in dynGENIE3 and we thus set *α*_*j*_ to the data-derived values (see the “Decay rate values” section). We also used these parameter values for Inferelator and Jump3, which also have degradation rates in their respective models. The resulting AUPR scores are shown in Table 1 and the AUPRs for each network are indicated in Supplementary Tables S2 and S3. A part of these results were taken directly from the work of Penfold & Wild^8^.

**Table 1:** AUPR scores of the DREAM4 networks learned from time series data.

Method	Algorithm	10-gene networks	100-gene networks
Tree ensembles	dynGENIE3	*4*.*410*	*47*.*596*
	GENIE3	1.915	13.635
	Jump3	3.610	43.434
Mutual information	tlCLR	4.006	39.020
	CLR	1.979	16.591
Dynamic Bayesian networks	G1DBN	3.705	24.186
	VBSSM	3.225	19.480
Ordinary differential equations	Inferelator	3.191	28.182
	TSNI	1.098	1.628
Non-linear dynamical systems	CSI	**4**.**733**	**57**.**543**
	GP4GRN	3.133	36.997
	OKVAR-Boost	0.762	3.868
Granger causality	GCCA	2.827	7.719
Random		0.260	0.643

The highest score is shown in bold and the runner-up is shown in italic. The scores of G1DBN, VBSSM, TSNI, CSI, GP4GRN and GCCA were computed from the AUPRs found in Tables 1 and 2 of Penfold & Wild^8^. The scores of Jump3 were computed from the AUPRs found in Tables 3 and S2 of Huynh-Thu & Sanguinetti^20^.

For each network size, dynGENIE3 is the second top-performing method, while GENIE3 returns much poorer predictions, stressing the importance of taking into account the dependence between the time points when exploiting time series data. The same effect is observed for CLR, with tlCLR performing better than its original counterpart. The best overall performer is CSI. This method however suffers from scaling issues (see the “Related works” section), while dynGENIE3 has a lower computational complexity and can thus be applied for the inference of very large networks.

#### Network inference from time series and steady-state data

We applied GENIE3 and dynGENIE3 for the joint analysis of time series and steady-state data (Table 2 and Supplementary Tables S4 and S5). dynGENIE3 yields the highest AUPR score when it integrates both datasets (compared to the scores obtained when only one of the two datasets is exploited), indicating that the two types of data contain different and complementary information that should be jointly exploited. GENIE3 returns here again poorer predictions than dynGENIE3. Its predictions for some networks are even worse than those obtained when only steady-state data are used.

**Table 2:** AUPR scores of the DREAM4 networks learned from steady-state and/or time series data.

Data	Algorithm	10-gene networks	100-gene networks
Steady-state	GENIE3	2.179	31.652
Time series	dynGENIE3	4.410	47.596
Steady-state + time series	GENIE3	2.542	30.884
Steady-state + time series	dynGENIE3	4.953	73.466
Knockouts	MCZ	4.428	98.9973
Knockouts + steady-state + time series	MCZ* dynGENIE3	5.983	**132**.**770**
	Challenge best performer	**7**.**085**	103.068

The highest score is shown in bold.

Two out of the three best performing methods of the DREAM4 *In Silico Network* challenge make an intensive use of the steady-state expression data resulting from the single gene knockouts^17,32^, highlighting the importance of this type of data for the inference of regulatory networks. To check if our dynGENIE3 procedure could be improved by an appropriate use of the knockout data, we combined it with the MCZ method^17^. In the latter procedure, the weight of the edge directed from gene *i* to gene *j* is given by the following median corrected *z*-score:

18 $${w}_{i,j}=\frac{|{x}_{i,ko}^{j}-{x}_{wt}^{j}|}{{\sigma }_{j}},$$

where ${x}_{i,ko}^{j}$ is the expression of gene *j* when gene *i* is deleted, ${x}_{wt}^{j}$ is the expected wild-type expression of gene *j*, and *σ*_*j*_ is the standard deviation of gene *j* expression. To combine MCZ with dynGENIE3, we simply take the product of the scores of the two methods. The final weight *w*_*i*,*j*_ will thus have a high value if the edge *i* → *j* is top-ranked by both methods. As shown in Table 2, the predictions of the networks can indeed be (strongly) improved when the two methods are combined. Actually, this MCZ/dynGENIE3 combination would have been ranked second and first in the 10-gene and 100-gene sub-challenges respectively. However, it requires a complete dataset comprising the systematic knockout of each gene of the targeted network, which may be unrealistic.

#### Influence of parameters

dynGENIE3 has two types of parameters: the model kinetic parameters *α*_*j*_, *j* = 1, …, *p* (decay rates of the genes) and the Random forest parameters (number *T* of trees per ensemble and number *K* of randomly selected variables at each tree node).

**Figure 1:**
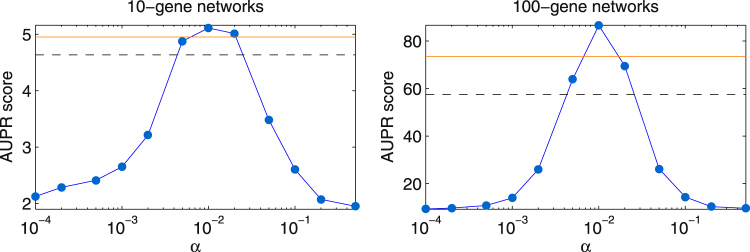
AUPR scores of the DREAM4 networks learned using dynGENIE3 on both the steady-state and time series data. Each dot corresponds to a case where all the decay rates *α*_*j*_ are set to the same value (indicated on the x-axis) and each horizontal line corresponds to a case where the *α*_*j*_ are respectively set to different values. The orange horizontal line is the score obtained when the *α*_*j*_ are set to the data-derived values and the dashed black horizontal line is the score obtained when the *α*_*j*_ are set to the decay rates that were used for the data simulation.

Figure 1 and Supplementary Figs S1 and S2 show that the quality of network predictions returned by dynGENIE3 highly depends on the values of the decay rates. The data-derived *α*_*j*_ values (orange horizontal line in the figures) yield a higher AUPR score than most of the other tested values of *α*_*j*_ and thus seem to be good default values for the inference of the DREAM4 networks. Setting *α*_*j*_ to the true decay rates, i.e. the decay rates that were actually used to simulate the DREAM4 data (these decay rate values were not available to the participants during the duration of the challenge), do not necessarily yield better performances (dashed black horizontal line in the figures). Actually, there is no clear correlation between the true and estimated decay rates (Supplementary Figs S3 and S4). The data-derived *α*_*j*_ thus only provide a rough order-of-magnitude estimate of the true decay rates, but nevertheless lead to good performance in terms of network reconstruction. This probably results from the mismatch between the dynGENIE3 model (7) and the model used for simulating the data, the decay rate values being adjusted to compensate for the fact that a different model is used.

Supplementary Table S6 shows the AUPR scores for different values of the Random forest parameters. The scores do not vary much when varying the number of trees. Although a drop in performance is observed when decreasing the value of *K*, the variations in the AUPR scores are much weaker here compared to the variations observed when varying the values of the kinetic parameters *α*_*j*_.

#### Predicting the network response to a double knockout

The DREAM4 challenge comprised a bonus round where the goal was to predict for each network the steady-state gene expression levels in several double knockout experiments (where two genes are simultaneously deleted). Given initial gene expression levels at *t* = 0, we used the ODE models learned by dynGENIE3 (from both the steady-state and time series data, using the data-derived decay rates) to predict the expression levels of non-deleted genes at successive time points until they reach a steady-state. The initial expression levels were set to zero for the two knocked out genes and to the wild-type expression levels for the remaining genes.

**Figure 2:**
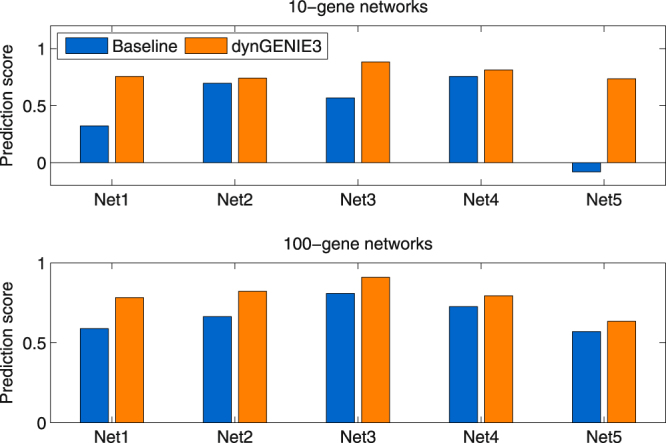
Predictive performances of dynGENIE3 and the baseline for the DREAM4 double knockout experiments. Each bar shows the Pearson linear correlation between the predicted and true expression levels, for all the double knockout experiments combined (5 experiments for each 10-gene network and 20 experiments for each 100-gene network).

We compared the (steady-state) predictions returned by dynGENIE3 to a baseline approach that uses the initial expression levels at *t* = 0 as predictions. For each network, our approach yields a higher correlation between the predicted and true expression levels than the baseline (Fig. 2). Although these correlation values are significant (Supplementary Table S7, where *p*-value < 1e-5 for all the correlation values), a large number of predictions remain however far from perfect (Supplementary Fig. S5).

### Real-world networks

We applied different network inference methods for the reconstruction of real-world sub-networks in three different organisms: *Saccharomyces cerevisiae*, *Drosophila melanogaster* and *Escherichia coli*. These organisms are much studied in the literature and known biology can hence be used here to guide the network inference. For each method and dataset, we restricted the candidate regulators to known transcriptions factors (TFs) and ranked all the putative regulatory interactions between these known TFs and the remaining genes. For dynGENIE3, Jump3 and Inferelator, we set the decay rate parameters to experimentally measured mRNA decay rates (see next section). For the genes for which a measured decay rate could not be retrieved, *α*_*j*_ was set to the median measured decay rate of the corresponding species.

#### Gene expression datasets

We used the following time series datasets (the numbers of samples, genes and TFs are indicated in Supplementary Table S1):

- *Saccharomyces cerevisiae* dataset^33^: This dataset comprises gene expression levels in the budding yeast, measured over 2 cell cycles in wild-type cells and 1.5 cell cycles in cyclin-mutant cells. To validate the network predictions, we used the gold standard network provided by the DREAM5 challenge^7^. We restricted our analysis to the genes that are periodically transcribed (as identified by Orlando *et al*.^33^) and that are also present in the gold standard. Measured mRNA decay rates were retrieved from the work of Geisberg *et al*.^34^.
- *Drosophila melanogaster* dataset^35^: This dataset comprises gene expression levels measured over the 24-hour period during which the embryogenesis of the fruitfly *D*. *melanogaster* takes place. We focused our analysis on the 1000 genes whose expression vary the most across the time series. We used as gold standard the experimentally confirmed binding interactions between TFs and genes that have been curated in the DroID database^36^ (version 2015_12). mRNA decay rates (measured from whole embryos) were retrieved from the work of Burow *et al*.^37^.
- *Escherichia coli* dataset^38^: This dataset comprises gene expression levels in *E*. *coli*, measured at several time points after five different perturbations: cold, heat, oxidative stress, glucose-lactose shift and stationary phase. We used as gold standard the verified regulatory interactions available in RegulonDB^39^ (version 9.0), and we focused our analysis on the genes that are present in both the dataset and the gold standard. mRNA decay rates (measured in cells with a growth rate of 0.63 h^−1^) were retrieved from the work of Esquerre *et al*.^40^.

#### Results

**Figure 3:**
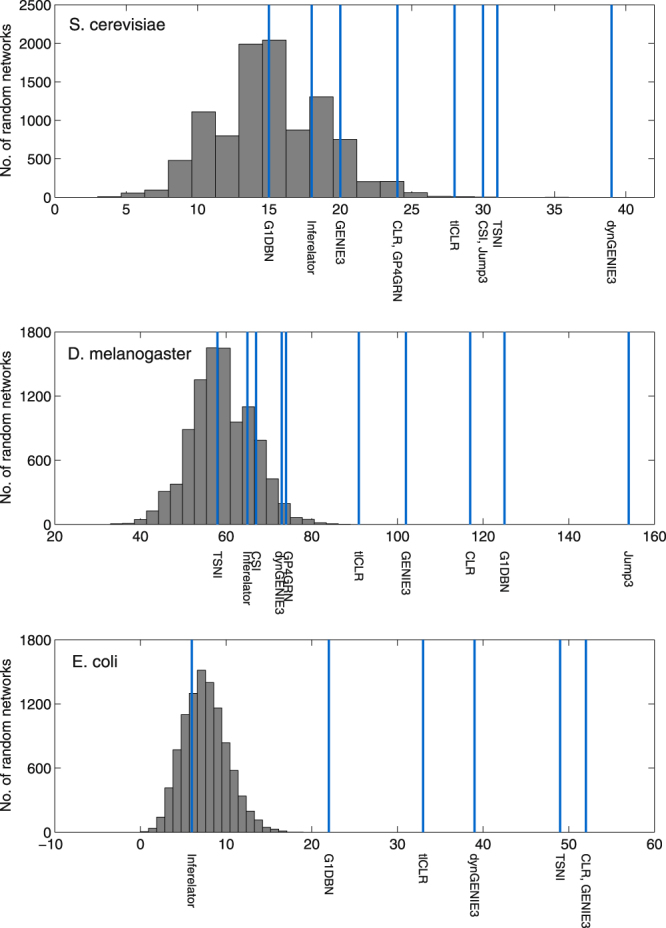
Inference of the *S*. *cerevisiae*, *D*. *melanogaster* and *E*. *coli* sub-networks. Each vertical line indicates the number of regulatory interactions that are shared between the top 500 edges predicted by one method and the gold standard network. The grey histogram shows the null distribution computed from 10,000 random networks. Due to their high computational complexities, Jump3, CSI and GP4GRN were not applied to the *E*. *coli* dataset.

Figure 3 shows for each organism the number of edges that are shared between the gold standard and the 500 regulatory links top-ranked by each method. To check if these numbers of shared edges are significant, we compared them to the numbers of edges that are shared between the gold standard and 10,000 random networks (represented by the grey histogram). The performances of the different methods depend very much on the organism. For example, while G1DBN is the second best performer for *D*. *melanogaster*, it does not perform better than random for *S*. *cerevisiae*. dynGENIE3, CLR and tlCLR are the only methods that retrieve a significant number of gold standard edges (*p*-value < 0.05) for each of the three organisms. The relative performances of GENIE3 and dynGENIE3 are also very data-dependent, with dynGENIE3 performing better than GENIE3 on the *S*. *cerevisiae* and DREAM4 datasets while the opposite is observed for *D*. *melanogaster* and *E*. *coli*.

As for the DREAM4 networks, the performance of dynGENIE3 on the real networks does not change much when using other values of the Random forest parameters (Supplementary Table S8), but strongly depends on the chosen values of the parameters *α*_*j*_ (see Supplementary Fig. S6). For *S*. *cerevisiae* and *E*. *coli*, setting *α*_*j*_ to the experimentally measured decay rates (black dashed horizontal line) allow to retrieve a high number of gold standard edges compared to the other tested *α*_*j*_ values. For *D*. *melanogaster*, although a significant number of true edges are retrieved with the experimentally measured decay rates, much better performances can be obtained with other values of *α*_*j*_. Supplementary Fig. S6 also shows that the data-derived values (orange horizontal line) yield reasonably good performances except in the case of *E*. *coli* where the top-500 edges do not contain any gold standard edge.

**Figure 4:**
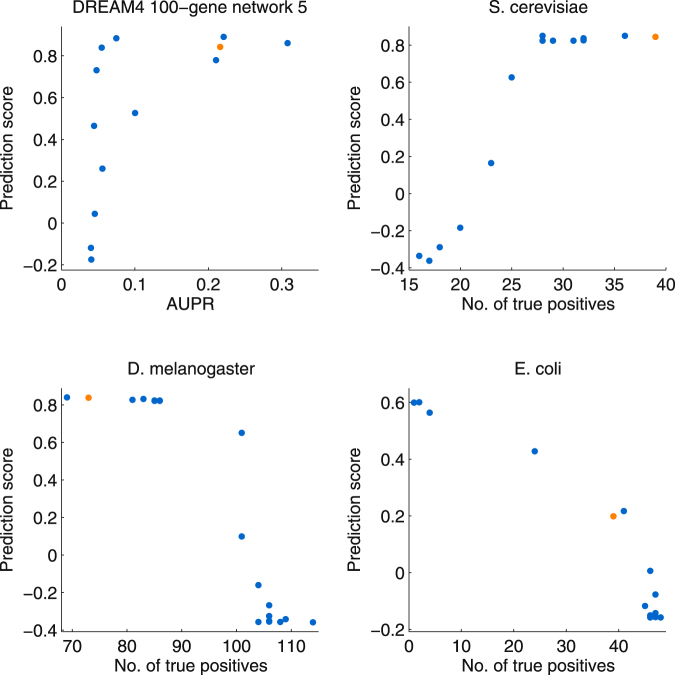
Performance of dynGENIE3 on one (representative) DREAM4 100-gene network and the three real-world networks. Each figure shows the correlation between the prediction score and the AUPR (for the DREAM4 network) or the number of retrieved gold standard edges among the 500 top-ranked edges (for the real networks). The predictions score is the Pearson linear correlation between the predicted expression levels *x*_*j*_(*t*_*k*+1_), ∀*k*, and the true levels in the out-of-bag samples, averaged over all the genes *j*. Each blue dot corresponds to a value of *α*_*j*_ (using the same *α*_*j*_ value $\forall j$). For the DREAM4 network the orange dot corresponds to the case where *α*_*j*_ are set to the data-derived values and for the real networks the orange dot corresponds to the case where *α*_*j*_ are set to the measured decay rates found in the literature.

It would thus be desirable to have an automatic way of tuning the kinetic parameters, which we first tried to achieve by checking how the ability of dynGENIE3 to predict new expression profiles vary according to the values of *α*_*j*_. One possible approach to get an unbiased estimate of the predictive performance of Random forest models is to use the out-of-bag samples, i.e. the samples that are left out when bootstrapping the original data before learning each tree. This approach has the advantage of being less computationally intensive than the usual cross-validation procedure. Using the out-of-bag samples, we measured the predictive performance of dynGENIE3 by computing the correlation between the predicted expression levels *x*_*j*_(*t*_*k*+1_) and the true ones. Figure 4 plots this prediction score versus the number of retrieved gold standard edges (or AUPR). Note that only one representative DREAM4 network is shown in the figure for the sake of space. The results obtained on the DREAM4 networks suggest that the prediction score allows the identification of the best values of *α*_*j*_, since a higher prediction score tends to coincide with a higher AUPR. However, this becomes less clear for the real networks, the prediction score being positively correlated with the number of retrieved edges for *S*. *cerevisiae* but negatively correlated for *D*. *melanogaster* and *E*. *coli*. Although disappointing, these results show that optimising the model predictive performance does not necessarily lead to a good feature selection (i.e. the selection of the true regulators for each target gene).

We also attempted to use a feature stability criterion^41^ in order to tune the parameters *α*_*j*_. The idea is to compare the *T* rankings of candidate regulators respectively returned by the *T* trees of an ensemble, the candidate regulators being each time ranked using the variable importance scores derived from one regression tree. More specifically, we used as stability score the average size of the intersection of the two sets of top 5 candidate regulators respectively returned by two regression trees:

19 $${\rm{stability}}\,{\rm{score}}=\frac{1}{p}\,\sum _{j=1}^{p}\,\frac{2}{T(T-\mathrm{1)}}\,\sum _{i=1}^{T}\,\sum _{i^{\prime} =i+1}^{T}\,\frac{\#({S}_{i,j}\cap {S}_{i^{\prime} ,j})}{5},$$

where *p* is the number of tree ensembles (one for each target gene) and *S*_*i*,*j*_ is the set of 5 top-ranked candidate regulators returned by the *i*-th tree of the *j*-th ensemble. Supplementary Fig. S7 plots this stability score as a function of the number of retrieved gold standard edges. Again, the results are not consistent over all the networks, as we do not observe a positive correlation for the *S*. *cerevisiae* network. On a general note, caution should however be taken when drawing conclusions from real data, since real gold standard networks are usually very far from being complete.

## Discussion

In this article, we evaluated the performances of tree-based approaches, GENIE3 and its dynamical variant dynGENIE3, for the inference of gene networks from time series of expression data. For this evaluation, we used artificial data from the DREAM4 challenge and real datasets related to three different organisms. Our experiments show that dynGENIE3 is competitive with diverse methods from the literature, even though it does not systematically yield the best performance for every network (but none of the compared methods was able to do so). Furthermore, our method can also be applied for the joint analysis of steady-state and time series data.

While dynGENIE3 consistently outperforms GENIE3 on the DREAM4 data, the same conclusion cannot be drawn for the real datasets, where the relative performances of the two methods are very data-dependent. These results could potentially be explained by the multiple differences that exist between the organisms and datasets, such as differences in the dynamics of the gene expression regulation or in the rates at which expression levels are sampled. A thorough analysis of these differences and their impact on the network inference methods would thus be desirable. As a preliminary result, Supplementary Table S9 shows the performance of dynGENIE3 when reducing by half the number of time points. Two different subsets of time points were used: the first half of the time points and the subset of time points obtained by taking every other time point over the whole time series. For the *D*. *melanogaster* and DREAM4 10-gene networks, most of the information seems to be contained in the first half of the time series, while for the other networks better performance is obtained when data are sampled over a longer time period.

As a side result, we showed that dynGENIE3 can be used to make predictions of gene expression profiles at successive time points. Here, we evaluated its predictive performances in the context of (simulated) double knockout experiments. Preliminary results show that dynGENIE3 performs better than a baseline approach. Such results should of course be completed with an evaluation on real data and a comparison to other predictive methods.

We investigated the predictive performance of dynGENIE3, estimated on the out-of-bag samples, as well as a stability criterion as means for automatically identifying the values of the kinetic parameters *α*_*j*_ that would yield the best performances in terms of network reconstruction. While both criteria appear to be good indicators for the artificial DREAM4 networks, they are not always positively correlated with the number of retrieved gold standard edges in the case of the real networks. The design of a method to automatically tune the parameters *α*_*j*_ is thus left as future work. Meanwhile, setting *α*_*j*_ to experimentally measured decay rates (or to the data-derived values when measured rates are not available) already allows to obtain good performances.

In our current implementation of dynGENIE3, we use the finite difference approximation to estimate the derivative $\frac{{\rm{d}}{x}_{j}(t)}{{\rm{d}}t}$ in the ODE model (7). Since this approximation relies on the time intervals between consecutive sampling time points, dynGENIE3 will miss the regulatory interactions that happen faster than the sampling frequency. Other approximation methods could be investigated, e.g. by computing the derivative of a Gaussian process fitted to the observed data *x*_*j*_(*t*_1_), …, *x*_*j*_(*t*_*N*_)^42^. Such a method would have the advantage of returning an estimate of the derivative at any time point *t* (and not only at the observation time points).

An important direction of future research is the application of the dynGENIE3 framework for the analysis of single-cell expression data. Emerging single-cell technologies now allow to measure gene expression levels simultaneously in hundreds of individual cells. Even when the gene expressions are measured at one single time point, cells are in different developmental stages, and several algorithms have been developed for ordering the cells along the developmental trajectory^43^. Pseudo time series derived from static single-cell data could therefore be used to unravel gene regulatory networks, and some promising initial steps are being taken^44^.

While we believe that dynGENIE3 is a step in the right direction, we also acknowledge that the complexity of gene regulation will pose a strict limit to the potential of GRN inference from expression data alone. Another important future research direction is thus the integration in dynGENIE3 of complementary data, such as microRNA expression, ChIP-seq, chromatin, or protein-protein interactions. Recently, Petralia *et al*.^45^, proposed an approach to bias the selection of features in Random forests, which could be adapted for dynGENIE3.

Like the Jump3 method, dynGENIE3 is a hybrid model-free/model-based method that incorporates a dynamical model within a non-parametric, tree-based approach. Various gene regulation models have been proposed in the literature, which could be exploited. These models differ in their level of details and also in the way they model uncertainties^46^. In the future, we plan to explore and evaluate other hybrid approaches combining parametric terms based on first principles with non-parametric terms in the form of tree ensembles.

## Electronic supplementary material

Supplementary information
