# Supplementary material for: dynGENIE3: dynamical GENIE3 for the inference of gene networks from time series expression data
Source: Sci Rep. 2018 Feb 21;8:3384. doi: 10.1038/s41598-018-21715-0 (PMC5821733; doi:10.1038/s41598-018-21715-0)
Supplement: Supplementary file 1 — Supplementary information [file 41598_2018_21715_MOESM1_ESM.pdf]

dynGENIE3: dynamical GENIE3 for the inference of gene networks from time series expression data
Supplementary information

Vân Anh Huynh-Thu [1] and Pierre Geurts [1]

[1]Department of Electrical Engineering and Computer Science
University of Liège, Liège, Belgium

1 Supplementary tables

Table S1: **Dataset information and running times of dynGENIE3 and CSI.** #TS: number of time series experiments, #TP: number of time points/ samples per time series experiment, N_{TS}: total number of samples in the time series dataset, N_{SS}: number of samples in the steady-state dataset, p: number of genes, r: number of candidate regulators (transcription factors), GEO: Gene Expression Omnibus (Edgar *et al.*, 2002). This table also shows the running times of dynGENIE3 and CSI when inferring the networks from the time series data only. These running times were measured on a 8GB RAM, Intel Core i7 2.5 GHz laptop. Due to its high computational complexity, CSI was not applied to the *E. coli* dataset.

	#TS	#TP	N_{TS}	N_{SS}	p	r	dynGENIE3	CSI
DREAM4 10 genes	5	21	105	31	10	10	9 sec	3 min
DREAM4 100 genes	10	21	210	201	100	100	6 min	200 days(*)
S. cerevisiae (GEO: GSE8799)	4	15	60	0	506	29	7 min	20 hours
D. melanogaster (GEO: GSE6186)	1	28	28	0	1000	14	11 min	5 hours
E. coli (GEO: GSE20305)	15	4-10	105	0	2006	163	90 min	-

(*) This running time was retrieved from Penfold and Wild (2011).

Table S2: **AUPRs of the DREAM4 10-gene networks learned from time series data.** The highest AUPR is shown in bold and the runner-up is shown in italic. The AUPRs of G1DBN, VBSSM, TSNI, CSI, GP4GRN and GCCA were taken from Table 1 of Penfold and Wild (2011). The AUPRs of Jump3 were taken from Table S2 of Huynh-Thu and Sanguinetti (2015). TE: tree ensembles; MI: mutual information; DBN: dynamic Bayesian networks; ODE: ordinary differential equations; NDS: nonlinear dynamical systems; GC: Granger causality.

Method	Algorithm	Net1	Net2	Net3	Net4	Net5
TE	dynGENIE3	*0.51*	*0.47*	**0.53**	**0.71**	0.71
	GENIE3	0.48	0.21	0.26	0.29	0.46
	Jump3	0.50	0.40	0.44	0.58	0.65
MI	tlCLR	0.47	0.45	0.41	0.56	**0.89**
	CLR	0.46	0.25	0.25	0.33	0.46
DBN	G1DBN	0.37	0.34	0.45	*0.69*	0.77
	VBSSM	0.38	0.41	*0.49*	0.46	0.64
ODE	Inferelator	0.44	0.43	0.42	0.52	0.56
	TSNI	0.27	0.32	0.21	0.23	0.25
NDS	CSI	**0.64**	**0.54**	0.45	0.67	*0.78*
	GP4GRN	0.42	0.44	0.47	0.35	0.65
	OKVAR-Boost	0.20	0.24	0.20	0.31	0.13
GC	GCCA	0.30	*0.47*	0.26	0.56	0.58
Random		0.17	0.18	0.17	0.14	0.13

Table S3: **AUPRs of the DREAM4 100-gene networks learned from time series data.** The highest AUPR is shown in bold and the runner-up is shown in italic. The AUPRs of G1DBN, VBSSM, TSNI, CSI, GP4GRN and GCCA were taken from Table 2 of Penfold and Wild (2011). The AUPRs of Jump3 were taken from Table 3 of Huynh-Thu and Sanguinetti (2015). TE: tree ensembles; MI: mutual information; DBN: dynamic Bayesian networks; ODE: ordinary differential equations; NDS: nonlinear dynamical systems; GC: Granger causality.

Method	Algorithm	Net1	Net2	Net3	Net4	Net5
TE	dynGENIE3	0.22	*0.14*	**0.25**	*0.22*	0.16
	GENIE3	0.05	0.06	0.10	0.06	0.09
	Jump3	**0.27**	0.11	0.20	0.18	*0.17*
MI	tlCLR	0.18	0.11	0.24	0.15	0.16
	CLR	0.08	0.06	0.12	0.07	0.09
DBN	G1DBN	0.11	0.10	0.13	0.10	0.11
	VBSSM	0.09	0.06	0.12	0.12	0.09
ODE	Inferelator	0.11	0.10	0.17	0.14	0.12
	TSNI	0.02	0.03	0.03	0.02	0.03
NDS	CSI	*0.25*	**0.17**	**0.25**	**0.24**	**0.26**
	GP4GRN	0.22	0.10	0.16	0.21	0.12
	OKVAR-Boost	0.05	0.05	0.03	0.02	0.02
GC	GCCA	0.04	0.04	0.07	0.07	0.03
Random		0.02	0.03	0.02	0.02	0.02

Table S4: **AUPRs of the 10-gene DREAM4 networks learned from steady-state and/or time series data.** The highest AUPR is shown in bold. SS: steady-state data, TS: time series data, KO: knockout data.

Data	Algorithm	Net1	Net2	Net3	Net4	Net5
SS	GENIE3	0.45	0.40	0.38	0.26	0.34
TS	dynGENIE3	0.51	0.47	0.53	0.71	0.71
SS + TS	GENIE3	0.51	0.30	0.36	0.38	0.48
SS + TS	dynGENIE3	0.64	0.52	0.62	0.61	**0.79**
KO	MCZ	0.80	0.28	0.69	0.71	0.39
KO + SS + TS	MCZ * dynGENIE3	0.82	**0.60**	0.80	0.77	0.59
	Challenge best performer	**0.92**	0.55	**0.97**	**0.85**	0.76

Table S5: **AUPRs of the 100-gene DREAM4 networks learned from steady-state and/or time series data.** The highest AUPR is shown in bold. SS: steady-state data, TS: time series data, KO: knockout data.

Data	Algorithm	Net1	Net2	Net3	Net4	Net5
SS	GENIE3	0.16	0.11	0.17	0.17	0.10
TS	dynGENIE3	0.22	0.14	0.25	0.22	0.16
SS + TS	GENIE3	0.11	0.12	0.19	0.13	0.14
SS + TS	dynGENIE3	0.34	0.22	0.32	0.34	0.22
KO	MCZ	0.48	0.38	0.38	0.36	0.17
KO + SS + TS	MCZ * dynGENIE3	**0.60**	**0.43**	**0.47**	**0.52**	**0.37**
	Challenge best performer	0.54	0.38	0.39	0.35	0.21

Table S6: **AUPR scores of the DREAM4 networks learned using dynGENIE3, for different values of the Random forest parameters.** These scores were obtained by learning the networks from the time series data only and from both the steady-state and time series data, with kinetic parameters α_j set to the data-derived values. SS: steady-state data, TS: time series data, T: number of trees per ensemble, K: number of input variables randomly selected at each tree node, n_{TF}: number of candidate regulators.

	10-gene networks		100-gene networks	
Data	TS	SS + TS	TS	SS + TS
$T = 100, K = n_{TF}$	4.457	5.142	46.110	71.580
$T = 200, K = n_{TF}$	4.427	4.987	46.619	72.061
$T = 500, K = n_{TF}$	4.404	4.937	47.342	73.536
$T = 1000, K = n_{TF}$	4.410	4.953	47.596	73.466
$T = 2000, K = n_{TF}$	4.392	5.022	47.577	73.441
$T = 5000, K = n_{TF}$	4.410	4.964	47.579	73.748
$T = 1000, K = \sqrt{n_{TF}}$	3.256	4.188	27.367	44.987
$T = 1000, K = n_{TF}/5$	3.111	4.083	30.992	51.110
$T = 1000, K = n_{TF}/2$	3.505	4.513	36.066	60.379

Table S7: **Pearson correlation between the true expressions and the expressions predicted by dynGENIE3, in the DREAM4 double knockout experiments.** For each network, the correlation coefficient was computed over all the genes in the different experiments combined. The corresponding p-values were estimated using 100,000 random permutations of the true expressions.

		Net1	Net2	Net3	Net4	Net5
10-gene networks	Correlation coeff.	0.756	0.740	0.882	0.813	0.735
	p-value	$< 1e-5$	$< 1e-5$	$< 1e-5$	$< 1e-5$	$< 1e-5$
100-gene networks	Correlation coeff.	0.783	0.823	0.909	0.794	0.635
	p-value	$< 1e-5$	$< 1e-5$	$< 1e-5$	$< 1e-5$	$< 1e-5$

Table S8: **Number of retrieved gold standard edges among the 500 edges top-ranked by dynGENIE3, for different values of the Random forest parameters.** These results were obtained by setting the kinetic parameters α_j to the mRNA decay rates retrieved from the literature. T: number of trees per ensemble, K: number of input variables randomly selected at each tree node, n_{TF}: number of candidate regulators.

	S. cerevisiae	*D. melanogaster*	*E. coli*
$T = 100, K = n_{TF}$	38	69	40
$T = 200, K = n_{TF}$	40	70	39
$T = 500, K = n_{TF}$	40	75	37
$T = 1000, K = n_{TF}$	39	73	39
$T = 2000, K = n_{TF}$	39	71	39
$T = 5000, K = n_{TF}$	38	73	39
$T = 1000, K = \sqrt{n_{TF}}$	39	78	41
$T = 1000, K = n_{TF}/5$	38	78	42
$T = 1000, K = n_{TF}/2$	41	75	39

Table S9: **Performance of dynGENIE3 when reducing by half the number of time points.** Given a time series with N time points $t_1, t_2, \ldots, t_N$, the performance of dynGENIE3 was assessed when inferring networks from the data at the following sets of time points: (1) Full time series: all the time points $(t_1, t_2, \ldots, t_N)$, (2) First half: the first half of the time points $(t_1, t_2, \ldots, t_{N/2})$, (3) Every two: half of the time points, obtained by taking every other time point over the whole time series $(t_1, t_3, t_5, \ldots, t_N)$. The performance is indicated in terms of AUPR score for the DREAM4 networks and in terms of number of retrieved gold standard edges among the 500 top-ranked edges for the real networks.

	Full time series	First half	Every two
DREAM4 10 genes	4.410	4.307	3.576
DREAM4 100 genes	47.596	32.197	41.222
S. cerevisiae	39	33	38
D. melanogaster	73	68	7
E. coli	39	17	33

2 Supplementary figures

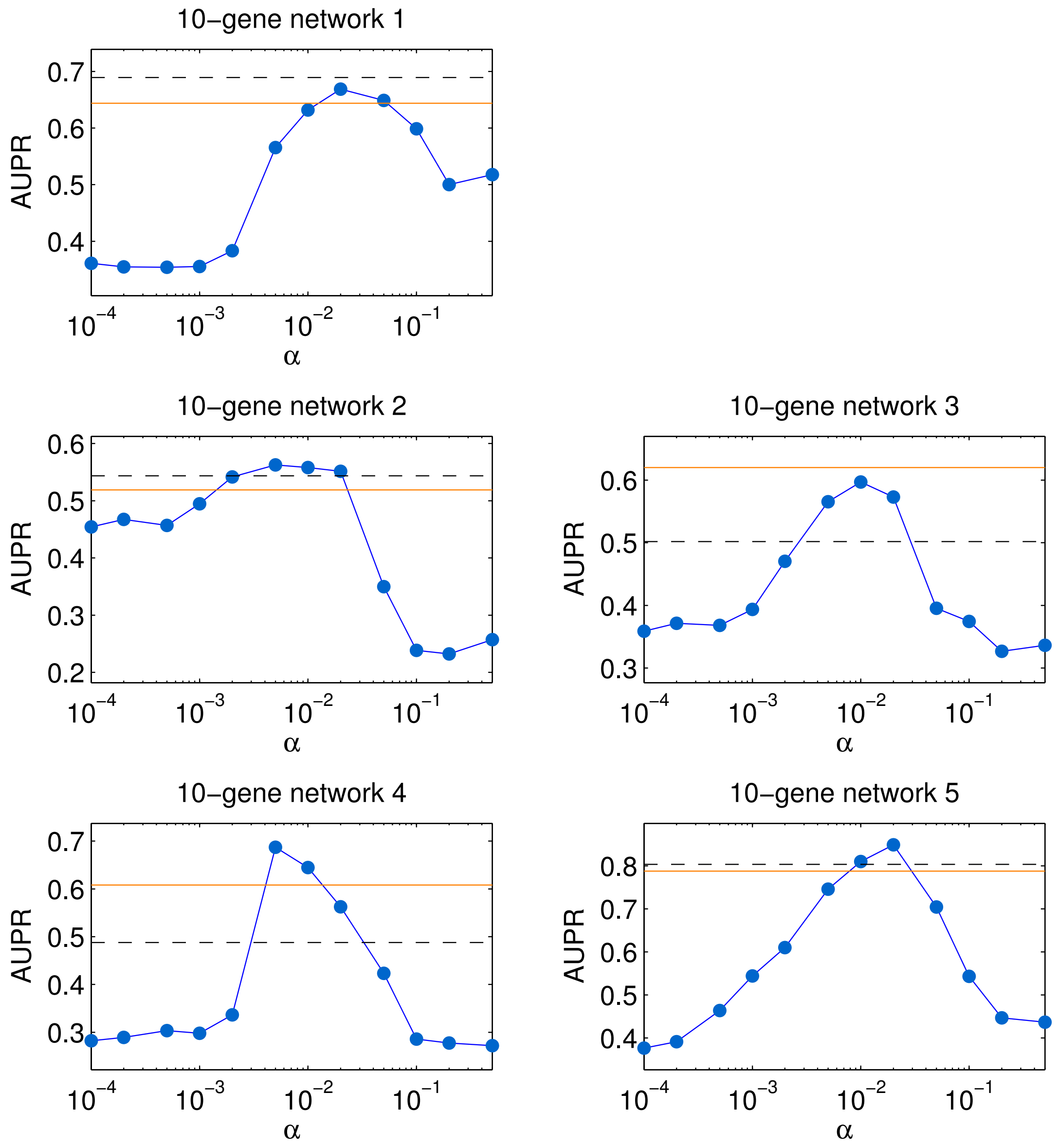

Figure S1: **AUPRs of the DREAM4 10-gene networks learned using dynGENIE3 on both the steady-state and time series data.** Each dot corresponds to a case where all the decay rates α_j are set to the same value (indicated on the x-axis) and each horizontal line corresponds to a case where the α_j are respectively set to different values. The orange horizontal line is the score obtained when the α_j are set to the data-derived values and the dashed black horizontal line is the score obtained when the α_j are set to the decay rates that were used for the data simulation.

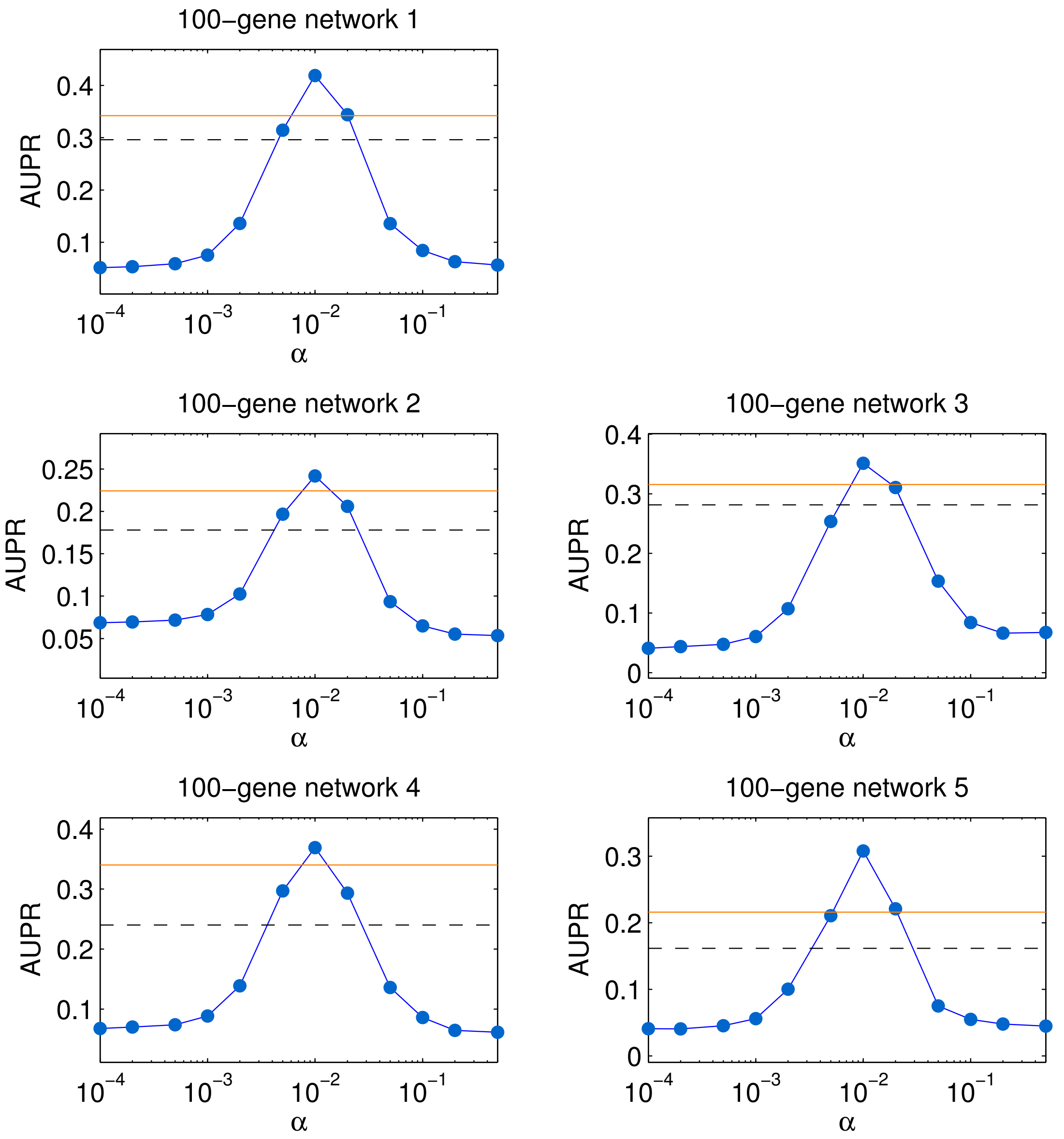

Figure S2: **AUPRs of the DREAM4 100-gene networks learned using dynGENIE3 on both the steady-state and time series data.** Each dot corresponds to a case where all the decay rates α_j are set to the same value (indicated on the x-axis) and each horizontal line corresponds to a case where the α_j are respectively set to different values. The orange horizontal line is the score obtained when the α_j are set to the data-derived values and the dashed black horizontal line is the score obtained when the α_j are set to the decay rates that were used for the data simulation.

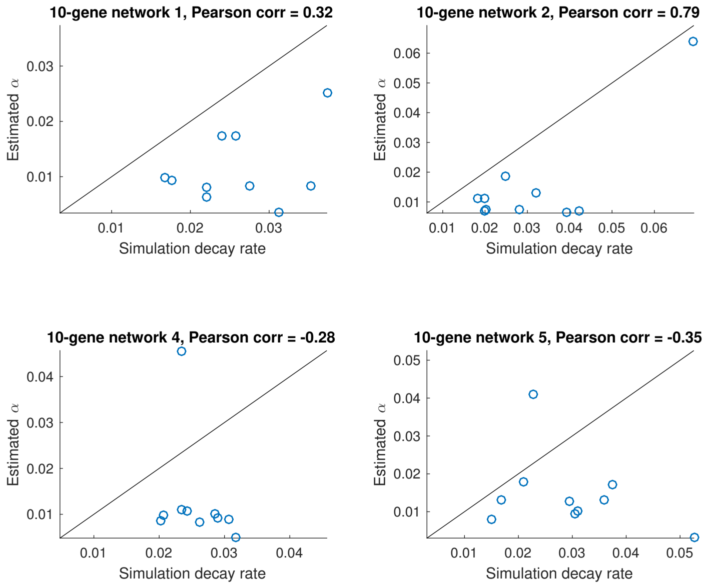

Figure S3: **Correlation between the decay rates used for simulating the DREAM4 size-10 data and the decay rates estimated using our method (data-derived values).** Each circle corresponds to one gene. The diagonal line indicates perfect matches.

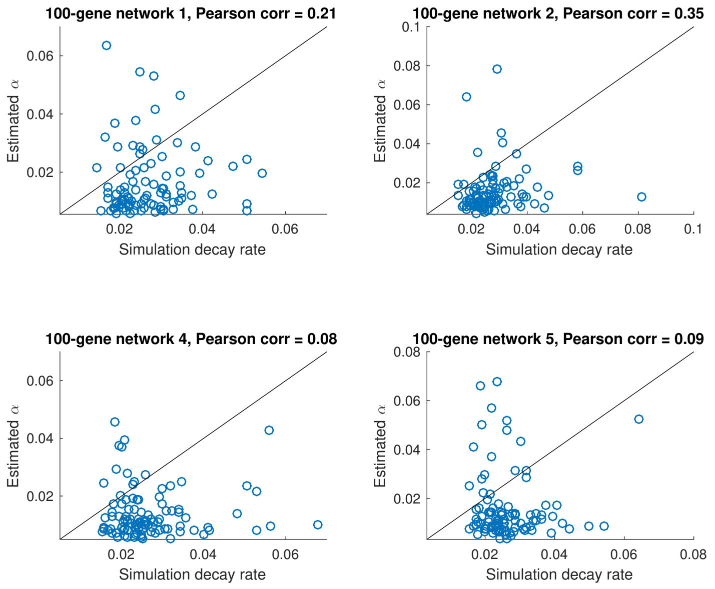

Figure S4: **Correlation between the decay rates used for simulating the DREAM4 size-100 data and the decay rates estimated using our method (data-derived values).** Each circle corresponds to one gene. The diagonal line indicates perfect matches.

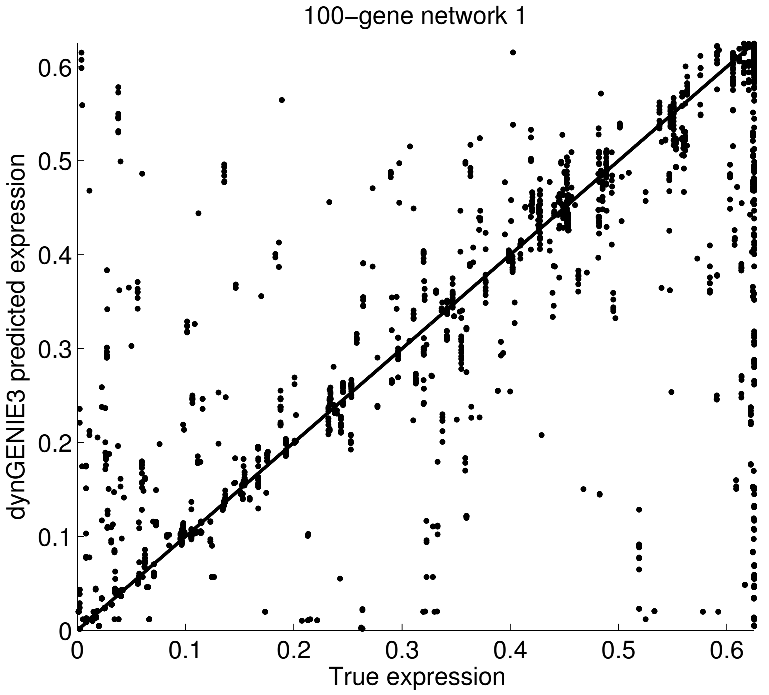

Figure S5: **Correlation between the true expressions and the expressions predicted by dynGENIE3, in the DREAM4 double knockout experiments.** The results shown here are those obtained for one DREAM4 100-gene network. Each dot corresponds to the prediction of the expression of one gene in one double knockout experiment. The diagonal line indicates perfect predictions.

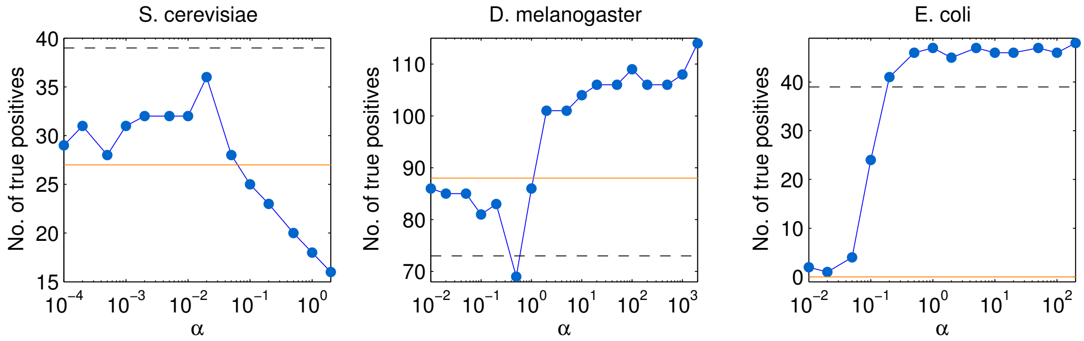

Figure S6: **Number of retrieved gold standard edges among the 500 edges top-ranked by dynGENIE3.** Each dot corresponds to a case where all the decay rates α_j are set to the same value (indicated on the x-axis) and each horizontal line corresponds to a case where the α_j are respectively set to different values. The black dashed horizontal line is the result obtained when the α_j are set to the experimentally measured mRNA decay rates retrieved from the literature and the orange horizontal line is the result obtained when the α_j are set to the data-derived values.

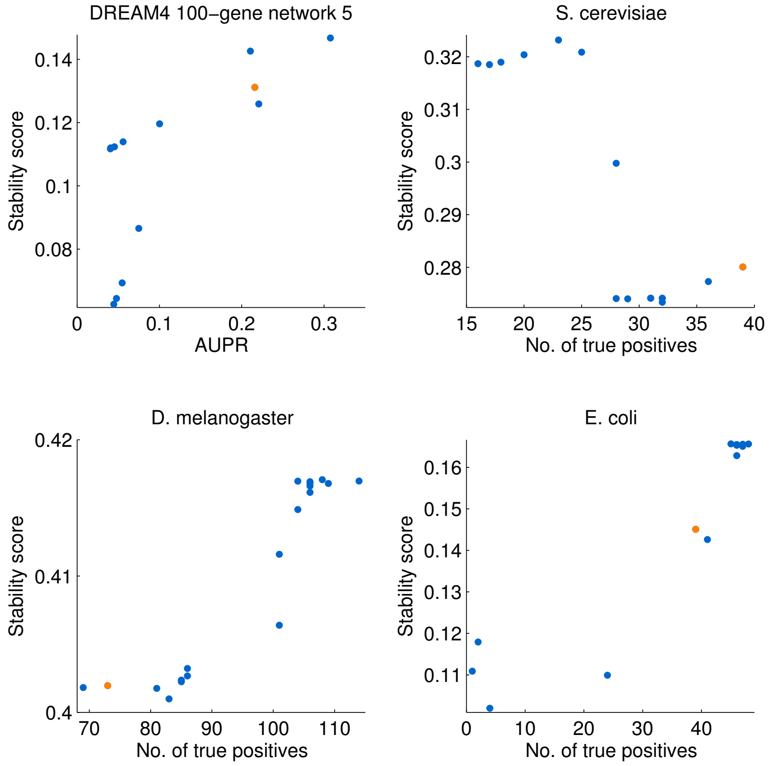

Figure S7: **Performance of dynGENIE3 on one (representative) DREAM4 100-gene network and the three real-world networks.** Each figure shows the correlation between the stability score and the AUPR (for the DREAM4 network) or the number of retrieved gold standard edges among the 500 top-ranked edges (for the real networks). The stability score is the average size of the intersection between the two sets of top 5 candidate regulators respectively returned by two regression trees. Each blue dot corresponds to a value of α_j (using the same α_j value $\forall j$). For the DREAM4 network the orange dot corresponds to the case where α_j are set to the data-derived values and for the real networks the orange dot corresponds to the case where α_j are set to the measured decay rates found in the literature.

References

Edgar, R., Domrachev, M., and Lash, A. E. (2002). Gene Expression Omnibus: NCBI gene expression and hybridization array data repository. *Nucleic Acids Research*, **30**(1), 207–210.

Huynh-Thu, V. A. and Sanguinetti, G. (2015). Combining tree-based and dynamical systems for the inference of gene regulatory networks. *Bioinformatics*, **31**(10), 1614–1622.

Penfold, C. A. and Wild, D. L. (2011). How to infer gene networks from expression profiles, revisited. *Interface Focus*, **1**(6), 857–870.
